# Maternal infection – but not inflammation – is associated with attention-deficit/hyperactivity disorder symptoms during childhood: a population-based cohort study

**DOI:** 10.1016/j.bbi.2025.106134

**Published:** 2025-10-17

**Authors:** Milan Zarchev, Frederieke A.J. Gigase, Lot de Witte, Charlotte A.M. Cecil, Manon H.J. Hillegers, Luz H. Ospina, Anna Suleri, Anna-Sophie Rommel, Ryan L. Muetzel, Veerle Bergink

**Affiliations:** a Department of Child and Adolescent Psychiatry/Psychology, Erasmus MC, Rotterdam, the Netherlands; b Department of Psychiatry, Erasmus MC, Rotterdam, the Netherlands; c The Generation R Study Group, Erasmus MC, Rotterdam, the Netherlands; d Department of Psychiatry, Icahn School of Medicine at Mount Sinai, New York City, NY, United States; e Department of Human Genetics, Radboud UMC, Nijmegen, the Netherlands; f Department of Psychiatry, Radboud UMC, Nijmegen, the Netherlands; g Department of Obstetrics and Gynecology, Erasmus MC, Rotterdam, the Netherlands; h Department of Radiology and Nuclear Medicine, Erasmus MC, Rotterdam, the Netherlands; i Department of Obstetrics, Gynecology and Reproductive Science, Icahn School of Medicine at Mount Sinai, New York City, NY, United States

**Keywords:** Infection, Inflammation, C-reactive protein, Cytokines, Fever, Maternal immune activation, ADHD, Neurodevelopment

## Abstract

**Background:**

Studies are equivocal on whether prenatal exposure to infection and inflammation is linked to higher risk of attention-deficit hyperactive disorder (ADHD) in the offspring. The current study investigated the relationship between prenatal exposure to maternal cytokines, C-reactive protein (CRP), fever and the risk of ADHD in childhood and early adolescence in the general pediatric population.

**Method:**

Data came from 6,555 mother–child pairs enrolled in Generation R, a Dutch prospective population-based cohort. The primary predictors were 1) self-reported fever and/or infections in each trimester; 2) a composite cytokine index of IL-1β, IL-6, IL-17a, IL-23, and IFN-γ and 3) CRP. The primary outcome was ADHD symptoms measured using the Child Behavioral Checklist (parent-reported) and Teacher Report Form (teacher-reported), obtained at three time points (ages 6, 10 and 14 years). Linear mixed models were used to estimate overall associations, adjusted for maternal factors (i.e., education, national origin, age, current psychopathology, psychosocial stress, smoking, pre-pregnancy BMI, parity) and child factors (i.e., age, sex, polygenic score for neurodevelopmental disorders). We also investigated if the associations were moderated by 1) the child’s polygenic score for neurodevelopmental disorders and 2) maternal stress during the pregnancy.

**Results:**

We found a small, but significant association between fever and ADHD symptoms (ß=0.06 [95 %CI = 0.01, 0.11]). No significant association was found between the cytokine composite index, individual cytokines, or CRP and ADHD symptoms. No interactions were detected for any of the exposures with a polygenic score for neurodevelopmental disorders (all interactions p-values > 0.198) or maternal stress (all p-values > 0.403).

**Conclusions:**

Our findings suggest that maternal fever and infections during pregnancy are linked to offspring ADHD symptomatology. The standardized effect size was smaller than that of other reported perinatal risk factors for ADHD. Cytokine levels and CRP as markers for chronic inflammation were not associated with ADHD.

## 1. Introduction

ADHD is a common familial neurodevelopmental disorder, for which multiple pre- and postnatal risk factors have been described (Han et al., 2021). Among these, there has been a clear interest in the role of infection and inflammation during pregnancy, given animal studies demonstrating a causal association with adverse neurodevelopmental outcomes in offspring (Brown & Meyer, 2018). However, there is substantial debate about how to investigate the association between infection, inflammation and ADHD in humans. While some researchers have stressed the importance of investigating inflammatory markers, such as C-reactive protein (CRP) and cytokines during pregnancy (Vázquez-González et al., 2023), others have focused on the acute inflammatory responses seen during bacterial or viral infections, especially if fever accompanies the infection (Le Gouez et al., 2016; Walle et al., 2022). These two research lines have produced results that are challenging to integrate into a clear public health message.

The impact of chronic low-grade inflammation during pregnancy has been investigated by measuring cytokines and CRP in maternal blood samples. Smaller studies recruiting between 62 and 293 children have shown conflicting findings (Smith et al., 2007). In some studies, higher levels of IL-6 during pregnancy were found to associate with children’s ADHD symptomatology at various ages (Gustafsson et al., 2020), while in others they were not (Thürmann et al., 2019). Even more puzzling, large population-based studies showed opposite findings with each other. Two Finnish registry studies in 1,079–5,127 children showed no association between prenatal CRP and offspring ADHD incidence (Chudal et al., 2020; Jallow et al., 2021). In contrast, one study of 700 mother–child pairs in Denmark found an extremely large odds ratio of 4 for the highest CRP quantile (>12.6 mg/L) and a clear dose–response relationship between maternal mid-pregnancy CRP levels and ADHD diagnosis at age 10 years (Rosenberg et al., 2024). In the same Danish cohort, exposure to a host of inflammatory markers during pregnancy, in particular IL-6, was associated with 50 % higher odds for inattentive symptoms at age 10 years, but not overall ADHD symptoms (Wang et al., 2025). The literature is thus considerably heterogenous on whether inflammation might be a significant and clinically relevant risk factor in the development of ADHD.

Besides maternal inflammation, the impact of infections during pregnancy has also been investigated. Maternal infections during pregnancy have been linked consistently to neurodevelopmental disorders in a systematic review of clinical studies (Antoun et al., 2021). However, a *meta*-analytic estimate for ADHD could not be obtained as it was the least studied neurodevelopmental condition. One population based study of 114,000 children showed a direct association between prenatal maternal fever and ADHD (Gustavson et al., 2019), while another only found an association specifically for genitourinary infections (Dreier et al., 2016). To date, no studies have looked at both maternal prenatal infections and inflammatory markers within the same cohort.

Moreover, there is a need to further elucidate potential mechanisms underlying the putative associations between prenatal exposure to infection and inflammation with ADHD, and to investigate which individuals are most susceptible (Hall et al., 2022). Not all maternal inflammatory insults lead to neurodevelopmental disorders in the offspring; inflammation could be a ‘second-hit’ on the causal pathway to adverse neurodevelopment (Han et al., 2021). According to this model, inflammation may interact with other risk factors such as stress and genetic liability for ADHD (Labouesse et al., 2015). In favor of this hypothesis, mice with certain genetic mutations have showed significantly more hyperactive behavior after prenatal exposure to inflammation compared to exposed mice with typical genetic profiles (Anderson et al., 2021). In addition, the combination of maternal stress and inflammation is hypothesized to produce larger associations with ADHD symptoms beyond their individual contributions (Howerton & Bale, 2012). Yet, no epidemiological study so far has provided empirical evidence for the role of genetic factors or maternal psychosocial stress as potential modifiers of the putative association between prenatal infection and inflammation and offspring ADHD.

To address these gaps, the current study investigated the association between maternal infection and inflammation during pregnancy and ADHD symptoms across childhood in a prospective general population cohort. We investigated a composite cytokine index, individual cytokines and CRP as markers of inflammation. Infection was defined as reported fever during the pregnancy (as a proxy for more severe infections), and additionally as a broader score of multiple common infections. Next, we investigated whether the associations between infection, inflammation, and ADHD symptoms were modified by the child’s polygenic risk for neurodevelopmental disorders and the mother’s psychosocial stress during pregnancy.

## 2. Methods

### 2.1. Population

This study was conducted within the Generation R cohort, a population-based cohort based in Rotterdam which included mothers with a delivery date between April 2002 and January 2006 (Kooijman et al., 2016). The current study included all Generation R mother–child dyads with at least one available measurement of blood serum cytokine (IL-1β, IL-6, IL-17a, IL-23, and IFN-γ) levels, C-reactive protein (CRP) levels, fever reported by the mother during pregnancy, and at least one assessment of ADHD symptoms via any informant/time point using the multi-informant ASEBA system (Achenbach & Rescorla, 2000, 2001). In case of sibling pairs, the sibling with the most complete data was included in the analysis. If equal amounts of data were available for both siblings, one sibling was chosen at random. Of the 9,901 women participating in the baseline measurement, 1,189 women did not have cytokine measurements and 18 did not have data for C-reactive protein. Moreover, 2,072 children did not have mother-reported Child Behavioral Checklist data at any time point. Finally, 67 siblings were excluded. The final analysis sample included 6,555 mother–child dyads.

### 2.2. Measures

#### 2.2.1. Inflammation

Maternal serum samples were collected at two time points in gestation at a median of 13.5 weeks (interquartile range: 10.8–16.2 weeks, n = 5,548) and a median of 20.6 weeks (interquartile range: 19.3–21.9 weeks, n = 6,800) through ante-cubital venous puncture. Blood samples were processed within three hours of collection, centrifuged for 10 min and immediately stored at – 80 °C. High Sensitivity (HS)-CRP was measured in EDTA plasma samples with an immunoturbidimetric assay, as described elsewhere (Ernst et al., 2011). Cytokines IL-1β, IL-6, IL-17a, IL-23, and IFN-γ were measured using the Human High Sensitivity T-Helper Cells Custom 5-plex assay from Millipore (Millipore, St. Charles, MO, USA) on the Luminex^™^ 100 system (Luminex, Austin, TX, USA) by Eve Technologies Corp. (Calgary, Alberta) in 2023. The selection of these immune biomarkers was based on a pilot study and existing literature (Gigase et al., 2024; Yockey & Iwasaki, 2018).

The pilot study (n = 100) was conducted to perform a data-driven selection from 14 cytokines (granulocyte macrophage–colony-stimulating factor, IL-1β, IL-2, IL-4, IL-5, IL-6, IL-8, IL-10, IL-12p70, IL-13, IL-17A, IL-23, IFN-γ, and TNF-α) (Suleri et al., 2025). In short, a panel of five cytokines (IL-1β, IL-6, IL-17a, IL-23, and IFN-γ) was selected based on high inter-cytokine correlations and a large range across the cytokines (IL-6 having higher sensitivity in the lower range and IL-23 in the higher range). In addition, the marker selection was based on the common assessment of CRP and IL-6 as low-grade inflammation markers, the potential role for the downstream effects of inflammation by IL-17 (Wong & Hoeffer, 2018) and to cover both the innate (IL-1β, IL-6, IL-23, IFN-γ) and adaptive T-cell (IL-17) immune response (Gigase et al., 2024). Cytokines showed moderate to high intercorrelations (r range = 0.40 – 0.86), enabling us to compute a cytokine composite index based on the averages of the two maternal serum measurements. The index comprised a summary score equal to the first principal component, which accounted for 66 % of total variance. Cytokines all loaded on the first component in the same positive direction. Cytokines were weakly correlated with CRP (r range = −0.06 – 0.09), which was examined as a separate exposure variable also averaged across the two timepoints. As described previously, inflammatory marker levels showed high variability between individuals and high temporal stability within individuals (Gigase et al., 2024).

#### 2.2.2. Infections

Data on prenatal infections were collected three times via a self-report questionnaire, once in each trimester of pregnancy. Mothers were asked to report on a variety of infections including a separate question at the end about whether they experienced a period of fever (>38 °C/100.4 °F) within the past 2 (second trimester) or 3 months (first and third trimester). A count variable was created ranging from 0 to 3 infection events (i.e., fever reported each trimester). A secondary measure of total infection based on the same questionnaire was also calculated. This total infection score included, in addition to fever, self-reported exposure to 1) any upper respiratory infections; 2) lower respiratory infections; 3) gastrointestinal infections; 4) cystitis/pyelitis; 5) dermatitis; 6) eye infections; 7) herpes zoster; 8) sexually transmitted diseases, and (9) the flu.

### 2.3. ADHD symptoms

#### 2.3.1. Child Behavioral Checklist (CBCL)

The mother-reported CBCL was repeatedly administered at the mean ages of 6 (n = 5,231), 10 (n = 4,119) and 14 years (n = 2,972) using the CBCL version 1.5–5 years for age 6 (56 % of children were still younger than 6 years old) and version 6–18 years for age 10 and 14 years. The CBCL version 1.5–5 years consists of 99 items, and the CBCL version 6–18 years consists of 112 items. Both versions are reliable and valid questionnaires widely used for assessing common emotional and psychiatric symptoms in children (Achenbach & Rescorla, 2000, 2001). ADHD symptoms were measured via the DSM-oriented ADHD problems scale, which includes 13 items such as “can’t sit still”, “impulsive”, and “can’t concentrate”. The scale has demonstrated adequate convergent validity with DSM-oriented ADHD psychiatric diagnoses obtained by clinical interviews in the general population (Aebi et al., 2010). ADHD symptoms were also reported by the father at age 10 years (n = 2,972) and by a teacher using the equivalent Teacher Report Form at age 6 years (n = 3,900). The continuous ADHD symptoms score from each time point and informant was converted to Z-scores to standardize across versions. Each measurement was included as a separate observation. For secondary analyses, each ADHD symptoms score was dichotomized as being above or below the top 5 % quantile cut-off, reflecting a conservative estimate of the prevalence of clinical ADHD symptoms in the general population (Ayano et al., 2023). In line with previous research on the current general population cohort, we chose this approach to ensure sufficient statistical power in the relevant sensitivity analyses (Levie et al., 2019).

#### 2.3.2. Conners’ Parent Rating Scale–Revised Short Form (CPRS-R)

The parent-reported CPRS-R was administered at age 8 years (n = 3,891) and was used as a separate secondary outcome. The questionnaire contains 27-items regarding the child’s hyperactive and impulsive behavior at home and other environments, of which 12 items form the ADHD index score were used in the current study. The ADHD index is designed to distinguish children with clinical levels of ADHD from typically developing children. Items are rated on a 4-point Likert scale (1 = not true at all; 2 = just a little true; 3 = pretty much true; 4 = very much true). The scale has been reported to have a good reliability and internal consistency in the Dutch population (Rijlaarsdam et al., 2015).

### 2.4. Moderators

#### 2.4.1. Polygenic score

A general polygenic score (PGS) for neurodevelopmental disorders was generated based on previous work (Hughes et al., 2023). Using gSEM, latent clustering of cross-disorder genomic data from eight psychiatric disorders (ADHD, Autism Spectrum Disorder (ASD), Tourette Syndrome (TS), Major Depressive Disorder (MDD), Anorexia Nervosa (AN), Obsessive Compulsive Disorder (OCD), Schizophrenia (SCZ), and Bipolar Disorder (BIP)) identified three factors: neurodevelopmental (ADHD, ASD, TS, MDD), compulsive (TS, AN, OCD), and mood-psychotic (MDD, BIP, SCZ). The current study used the neurodevelopmental factor score, reflecting overall genetic susceptibility for neurodevelopmental disorders. A liberal p-value threshold of 1.0 was applied for the construction of the PGS, consistent with prior studies linking PGSs to child behavior outcomes.

#### 2.4.2. Maternal psychosocial stress

A cumulative prenatal stress score based on previous work was used (Defina et al., 2024). The score summed prenatal events occurring in two domains: (a) life events (e.g. unplanned pregnancy, death of a loved one) and (b) interpersonal risk (e.g., family conflicts, divorce). All stress indicators were dichotomized into ‘risk’ (1) or ‘no risk’ (0) and the mean averaged to form the scores for each domain. The cumulative score for prenatal stress was then computed by summing its respective domain scores.

Information was further collected on maternal acetaminophen use during pregnancy. Mothers were asked at each trimester to retrospectively self-report if they have taken acetaminophen at any point from conception to week 32. Mothers were classified as having taken vs. not having taken acetaminophen at any point during pregnancy.

### 2.5. Covariates

Self-report questionnaires on enrollment were used to assess maternal age (continuous), education (categorized as primary, secondary or higher), national origin (Dutch, other Western or non-Western), smoking (no, smoked until pregnancy was known, continued smoking during pregnancy), parity (continuous) and pre-pregnancy BMI (continuous, kg/m^2^). Current maternal psychopathology at enrollment was measured using the self-report Brief Symptom Inventory (BSI), specifically using the global severity index (continuous) which combines multiple symptom domains into an overall psychopathology score (Derogatis & Melisaratos, 1983). The child’s biological sex at birth was obtained via medical registers. Child age (continuous) was calculated at each measurement wave from the child’s birthday.

### 2.6. Statistical analysis

We used linear mixed-effects models to investigate the associations of prenatal inflammation and infection with child ADHD, measured at repeated time points and across informants. First, the three exposures of interest were investigated using three separate regression models focusing on 1) the cytokine composite index; 2) CRP level and 3) self-reported fever. The primary outcome was CBCL ADHD symptoms scores measured at ages 6, 10 and 14 years. We adjusted all models for parental factors that could confound the associations between inflammation, infection and ADHD symptoms, namely maternal education, national origin, maternal age, current maternal psychopathology, maternal psychosocial stress during pregnancy, smoking, pre-pregnancy BMI, and parity and for the child’s polygenic score for neurodevelopmental disorders (Supplementary Fig. 1). Child sex and age at ADHD assessment were also included as precision covariates. To obtain time-specific and informant-specific estimates, the models included a three-way interaction term between 1) the exposure; 2) the measurement wave and 3) the informant providing the observation. A random intercept and slope for time was included for each participant. We applied a false discovery rate (FDR) correction for multiple testing of the three exposures.

The primary estimate of interest was the *overall* associations between inflammation/infection and ADHD symptoms. These overall associations were estimated using marginal means averaged across measurement waves and informants. Time-specific (age 6, 10 and 14 years) and informant-specific (mother, father, teacher) associations were also calculated using estimated marginal means. Models were fit using the “lme4” packages and marginal means calculated using the “emmeans” package in R v.4.1 (R Core Team, 2021). The analysis plan and preregistered code for this project are available via timestamped files at https://osf.io/84wnm/.

Secondly, an identical strategy was used, except the model included only a two-way interaction consisting of 1) the exposure and 2) each moderating factor (i.e., PGS of neurodevelopmental disorders or maternal psychosocial stress). Informant and measurement were included as fixed-effect covariates. Each combination of inflammation/infection exposure and moderating factor was analyzed in separate linear mixed models. Marginal means were again used to estimate the associations between inflammation/infection and ADHD symptoms in the top and bottom quintile of the moderating factor. P-values were obtained from the two-way interaction coefficients.

### 2.7. Sensitivity analyses

A set of sensitivity analyses probed the overall associations between inflammation, infection and ADHD symptoms. First, the primary CBCL ADHD outcome was replaced with a secondary outcome consisting of the CPRS-R ADHD index. Separate linear regressions were run between each exposure and the CPRS-R, which was only available at age 8 years. Second, the dimensional ADHD scores were dichotomized into “clinical” vs. “subclinical” categories for each time point. We ran a logistic mixed-effects models to estimate the association between the exposures and a research diagnosis of ADHD. To obtain overall associations from the logistic mixed-effects model, we report on overall odds ratios marginalized over all ADHD measurements using estimated marginal means. Third, we investigated the timing of inflammation by replacing the averaged cytokine and CRP levels with measurements obtained in the second trimester of pregnancy (~13 weeks; 95 % range = 9.6–17.6 weeks) or later in mid-pregnancy (~20 weeks; 95 % range = 18.5–23.3). Fourth, we investigated the timing of infection by looking at trimester specific association between fever and ADHD symptoms. Fifth, we investigated individual cytokine associations with ADHD symptoms to further investigate the association with individual inflammatory markers. Sixth, we estimated separate sex-specific associations for boys and girls. Seventh, the fever exposure was expanded to include multiple types of infections comprising 1) any upper respiratory infections; 2) lower respiratory infections; 3) gastrointestinal infections; 4) cystitis/pyelitis; 5) dermatitis; 6) eye infections; 7) herpes zoster; 8) sexually transmitted diseases, and (9) flu. Eight, we re-ran moderation analyses with the neurodevelopmental PGS on a subpopulation of children from European background to ensure ancestry did not impact the results. Ninth, we investigated acetaminophen use during pregnancy as a potential moderator, presenting each association separately for users and non-users.

### 2.8. Missing data

Covariate data was missing for maternal factors, i.e., psychosocial stress (24.9 %), psychopathology at enrollment (19.0 %), pre-pregnancy BMI (17.3 %), smoking during pregnancy (10.8 %) and maternal educational level (6.3 %) and the child’s neurodevelopmental PGS (13.5 %). Missing data was imputed using multiple imputation chained equations (40 imputed datasets, 100 iterations) as implemented in the “mice” R package. We imputed all missing covariate and outcome data for participants who meet the inclusion criteria, in line with current recommendations (Enders, 2022). Participants in the final analysis sample were compared to those not in the analysis in Supplementary Table 1. On average, mothers included in the analysis tended to be older (mean age 30.2) compared to those not in the analysis (29.2), were more likely to be Dutch (53 % vs 34 %), highly educated (46 % vs 35 %) and were less likely to have a pre-existing mental illness (14 % vs. 23 %). The cytokine levels of participating mothers were slightly higher than non-participating ones (Cohen’s d range 0.04–0.10), with no difference observed for IL6. CRP was slightly lower in participating mothers (Cohen’s d = 0.10) and no differences were observed for fever and the infection score.

## 3. Results

The final sample comprised a total of 6,555 mother–child pairs. Sample characteristics are presented in Table 1, stratified by whether children were in the top 5 % of ADHD symptoms at any measurement point.

**Fig. 1.**
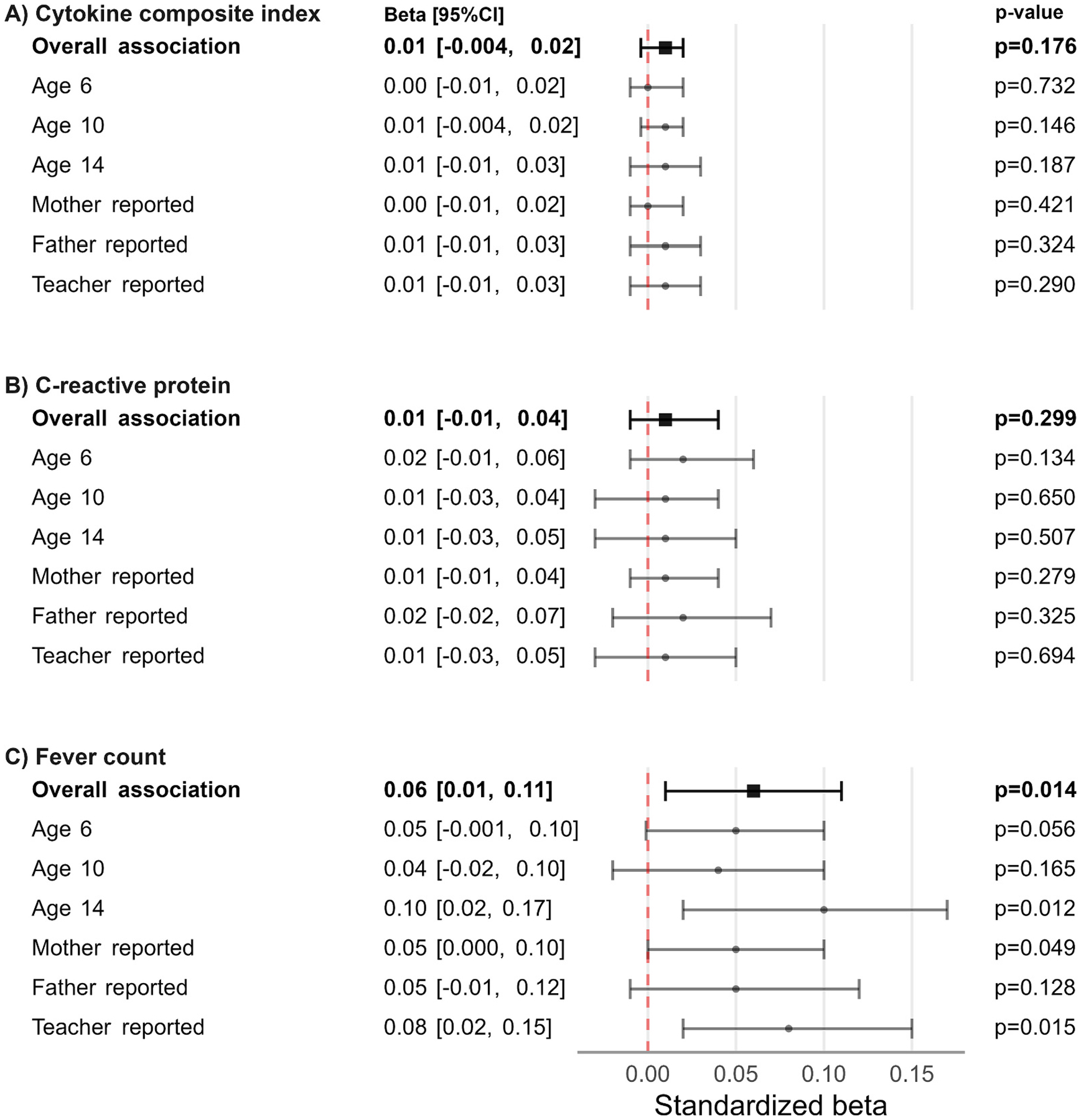
Overall, age-specific, and informant-specific standardized associations between maternal inflammation/infection and ADHD symptoms. Overall associations represent weighted averages marginalized over all time and informant assessments. Note: All estimates adjusted for maternal age, maternal education, maternal national origin, maternal psychopathology, maternal stressful events during pregnancy, maternal smoking during pregnancy, maternal body mass index before pregnancy, parity, child polygenic risk score for neurodevelopmental disorders, child age and child sex.

The covariate adjusted associations are presented in Fig. 1. After adjustment, the maternal cytokine composite index (ß = 0.01 [95 %CI = – 0.004, 0.02], p = 0.176) and CRP (0.01 [−0.01, 0.04], p = 0.299) were not significantly associated with child ADHD symptoms. In contrast, self-reported prenatal fever was associated with child ADHD symptoms (0.06 [0.01, 0.11], p = 0.014), indicating each additional count of maternal fever exposure corresponded to a 0.06 standard deviation increase in the standardized ADHD symptoms score. The association remained significant after multiple testing correction for three exposures (adjusted p = 0.042). The effect sizes were of similar magnitude across time points and informants. Unadjusted associations between the cytokine composite index, CRP, fever and ADHD symptoms are presented in Supplementary Fig. 2. Of note, the unadjusted associations between CRP and ADHD symptoms were significant across all informants and time points.

**Fig. 2.**
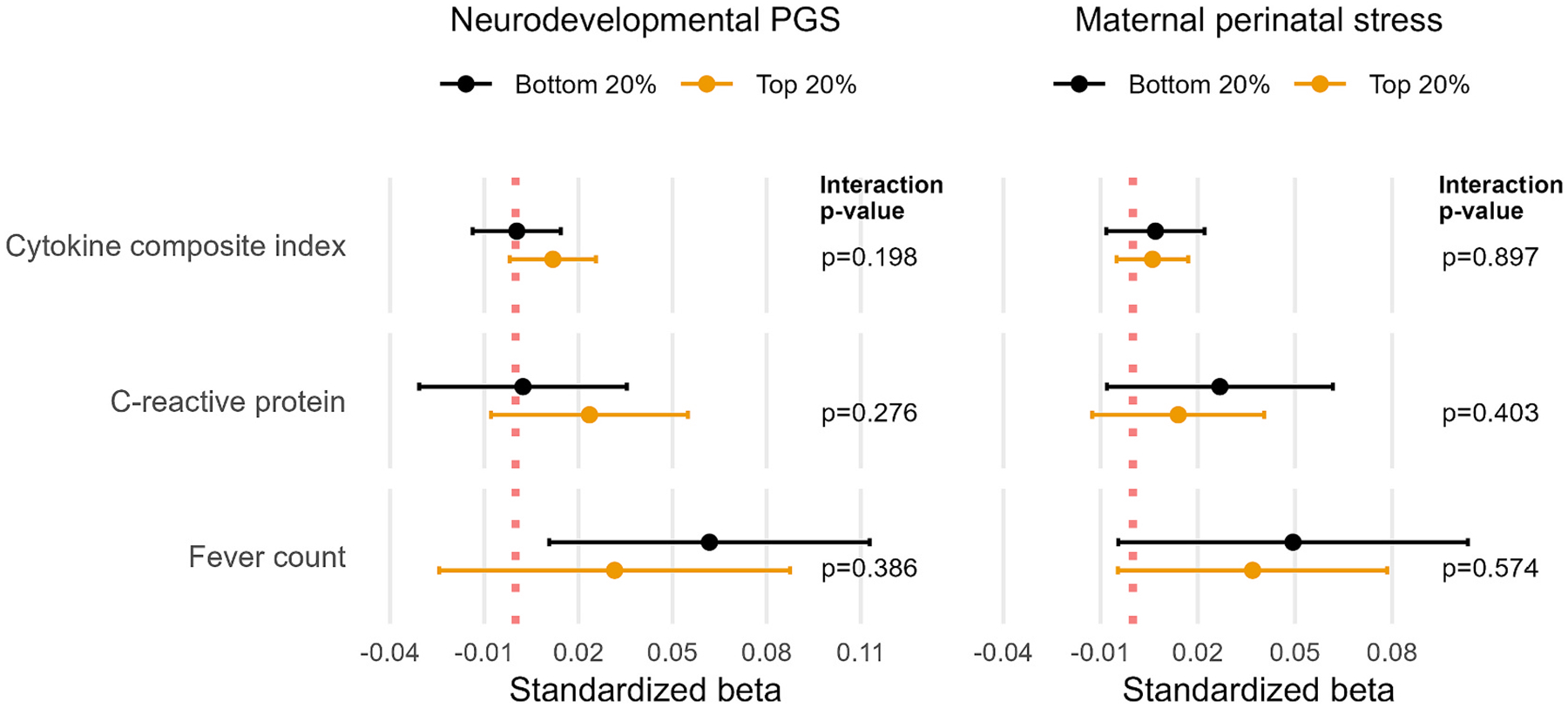
Associations between maternal inflammation/infection and ADHD symptoms moderated by genetic risk and maternal prenatal stress. Each point corresponds to standardized beta coefficients, stratified by moderator quantile (e.g. bottom 20 % genetic risk quantile in black vs. top 20% genetic risk quantile in orange). P-values obtained from interaction term between inflammation/infection exposure and moderator. Note: All estimates adjusted for maternal age, maternal education, maternal national origin, maternal psychopathology, maternal smoking during pregnancy, maternal body mass index before pregnancy, parity, child age and child sex.

Secondly, we investigated if the overall associations between the inflammation/infection exposures and ADHD symptoms were moderated by the neurodevelopmental polygenic score or by maternal prenatal stress presented (Fig. 2). We found no evidence that the PGS (all interactions p-values > 0.198) or the maternal prenatal stress score (all interactions p-values > 0.403) moderated any of the associations.

As sensitivity analyses, we first replaced the primary CBCL ADHD symptoms outcome with the parent-reported CPRS-R at age 8 years. There were no significant associations with any of the three inflammation/infection exposures (all p-values > 0.204; Supplementary Table 2). Second, we found no significant associations between prenatal inflammation/infection with clinical and subclinical ADHD groups (all p-values > 0.467; Supplementary Table 3). Third, we found similar point estimates for the cytokine composite index and CRP associations, regardless of whether they were measured early in the second trimester (~13 weeks) or later in mid-pregnancy (~20 weeks; Supplementary Fig. 3). Fourth, point estimates were also comparable for the associations of fever in the first, second and third trimester with child ADHD symptoms (Supplementary Table 4). Fifth, individual prenatal cytokine levels (i.e., IL-1β, IL-6, IL-17a, IL-23, and IFN-γ) were not significantly associated with child ADHD symptoms as measured by the CBCL or CPRS-R (all p-values > 0.182; Supplementary Table 4). Sixth, we found no evidence for child sex moderating any of the associations between inflammation/infection and ADHD symptoms (all interaction p-value > 0.333, Supplementary Fig. 4). Seventh, the association between prenatal infections and child ADHD symptoms was also significant when the fever exposure was replaced with a broader infection score consisting of a range of viral and bacterial infections during pregnancy (standardized beta = 0.02 [0.003, 0.03], p = 0.010; Supplementary Fig. 5), indicating each additional maternal infection corresponded to a 0.02 standard deviation increase in the standardized ADHD symptoms score. Eight, interaction with the neurodevelopmental PGS remained non-significant if focusing only on children from European background (all interaction p-value > 0.295; Supplementary Fig. 6). Ninth, there were no significant differences in the inflammation/infection associations between mothers who did and did not take acetaminophen during pregnancy (all interaction p-value > 0.278; Supplementary Fig. 7).

## 4. Discussion

The current study investigated the association between maternal inflammation and infections during pregnancy, and ADHD symptoms across childhood and early adolescence in a large population-based longitudinal cohort of mother–child pairs. We found no evidence for associations of prenatal exposure to cytokines (IL-1β, IL-6, IL-23 or IFN-γ) or CRP with parent- and teacher-reported ADHD symptoms across ages 6 to 14 years. However, an association was found between prenatal fever at any point in pregnancy (as a proxy for more severe infections) and child ADHD symptoms. Similarly, common infections during pregnancy were significantly associated with child ADHD symptoms. We found no evidence that these associations were moderated by the child’s polygenic score for neurodevelopmental disorders, or maternal prenatal psychosocial stress. We observed no effect of timing of infection, inflammation or child sex on these associations.

Prenatal infection was associated with symptoms of ADHD in the child, regardless of whether infection was defined by fever or a broad range of common infections. Our findings are in line with prior studies in humans showing increased ADHD symptom scores after maternal infection during pregnancy, albeit with small effect sizes (Antoun et al., 2021). Previously, one study raised concern that the association between maternal infection and ADHD might be confounded by genetic and socio-environmental factors (Ginsberg et al., 2019). Using a sibling-matched design, they found that the association with ADHD diagnosis lost significance when discordant sibling pairs were considered. However, the effective sample size of ADHD cases within sibling pairs was less than 260 children and thus the study might have lacked statistical power to detect small associations. This is in line with our non-significant sensitivity analyses of the associations of fever during pregnancy with a research diagnosis of ADHD or ADHD symptoms measured at a single time point, which had reduced power to detect smaller effects. Nevertheless, larger cohorts continued to detect a small association between prenatal exposure to infection and child ADHD after statistically adjusting for polygenic ADHD scores or family history of mental illness (Leppert et al., 2019; Walle et al., 2022). It is also worth noting that maternal medication use as a consequence of infection, particularly acetaminophen, has been reported to be independently associated with ADHD symptoms, including in the current cohort (Alemany et al., 2021; Zhu et al., 2022). In a sensitivity analysis we found no evidence the associations reported were different amongst mothers who did or did not use acetaminophen during pregnancy. Similarly, one large study (n = 99,947) specifically looked at maternal fever in combination with acetaminophen use and found no difference in association with children’s ADHD diagnosis among those who did or did not take medication (Gustavson et al., 2019). The standardized effect sizes between prenatal exposure to infection and child ADHD symptoms were marginally smaller than the reported *meta*-analytic standardized effect sizes of other ADHD perinatal risk factors such as a low birthweight, preterm birth, or a low Apgar score (Bitsko et al., 2024). Taken together, our study and others suggest that maternal fever and infections during pregnancy are associated with marginally increased ADHD symptom scores across childhood and early adolescence.

In contrast to smaller clinical studies (n < 300) (Graham et al., 2018; Gustafsson et al., 2020; Thürmann et al., 2019), we did not find an association between mid-pregnancy prenatal cytokines and ADHD symptoms. While these studies focused on mid- to late pregnancy as well, they did not adjust for confounders such as pre-pregnancy BMI and background socioeconomic factors which may explain differential findings. In our study we demonstrated that, prior to adjusting for key confounders, a highly significant yet likely spurious correlation between maternal CRP and childhood ADHD symptoms emerged. Additionally, our results are supported by a population-based Danish cohort (n = 700), which after adjustment for confounders reported no association between a cytokine composite (18 inflammatory markers) in mid-pregnancy and child ADHD or symptoms at age 10 (Wang et al., 2025). The same Danish study did find an association, however, with only inattentive symptoms. These findings suggest that in larger population-based cohorts, the association between prenatal cytokines and ADHD symptoms might not replicate readily.

In line with two large Finnish cohorts (n = 1,079–5,127) (Chudal et al., 2020; Jallow et al., 2021), we did not find an association between prenatal CRP levels and child ADHD symptoms. However, the aforementioned population-based Danish cohort also investigated the association between maternal CRP and ADHD in children. A highly precise dose–response relationship was found between each quantile of CRP in late pregnancy and overall ADHD diagnosis (Rosenberg et al., 2024) The reported effect size in the highest quintile of CRP was an odds ratio of 4, considerably larger than biggest established risk factors for ADHD in the perinatal period (e.g., emergency delivery, extremely low birth weight, neonatal intensive care) (Bitsko et al., 2024; Serati et al., 2017). The authors address the discrepancy in results with the aforementioned Finish cohorts by referring to 1) differences in timing of the CRP measurement (early vs. mid-pregnancy) and 2) their deep phenotyping of ADHD, including via dimensional symptom scores. Our null results are opposite to prior Danish findings, which is remarkable given that we measured inflammation markers during a similar period of pregnancy and adjusted for similar confounders. As mentioned before, the Danish cohort did collect clinical diagnoses of ADHD, which could tap into symptomatology beyond the parent and teacher reported behaviors measured in the current study. However, the same study also reported strong associations with a parent-reported ADHD dimensional score comparable to the current ADHD outcome, albeit measured via a different instrument. We therefore tentatively conclude there is no strong evidence that prenatal CRP is a clinically significant risk factor for later ADHD symptoms in children, with one notable exception in the literature.

We attempted to model genetic susceptibility and maternal psychosocial stress factors that have been hypothesized to interact with prenatal inflammation on the pathway to child ADHD symptoms, but found no evidence for prenatal inflammation or infection acting as ‘second-hits’. Heritability is high for ADHD (h^2^ = 0.70–0.80), which could provide basis for why no additional variance could be explained by the interactions (Brikell et al., 2015; Suleri et al., 2023). It is further possible that the mechanisms through which prenatal infection might impact child ADHD symptoms (e.g. direct pathogenic effects; al-Haddad et al., 2019) are non-cumulative and independent from the mechanisms through which genetic vulnerability and perinatal psychosocial stress operate.

## 5. Strengths and limitations

A major strength of the current study is the ability to address a comprehensive range of known socioeconomic, clinical and genetic confounders (Dreier et al., 2016). Yet, while the adjustment strategy was more comprehensive than previous studies, residual confounding cannot be ruled out completely, especially residual genetic confounding not captured by the neurodevelopmental PGS. On the exposure side, the temporal reliability and convergent/discriminant validity of the cytokine and CRP values were extensively validated before they were used in the current study (Gigase et al., 2024). Imperfect measurement of infection (e.g. recall bias) and inflammation (measured twice in mid-pregnancy), however, could still result in attenuated associations. Fever may also have resulted from non-infectious causes like autoimmune disorders (Mulders-Manders et al., 2015). A further limitation is that we cannot relate spikes of inflammation to concrete infection exposures, as the half-lives of cytokines and CRP are relatively brief (Ho & Lipman, 2009). Mothers are unlikely to visit the research center during that brief interval of heightened inflammation, when infection symptoms are at their worst. On the outcome side, a key strength of the study was obtaining measurements from multiple informants at multiple time points, including from fathers and teachers. This helps to address shared reporter bias arising from mothers reporting on both infection during pregnancy and their children’s ADHD symptoms. Additionally, teacher assessments are particularly valuable for measuring out of home ADHD symptoms, as teachers are able to directly compare children to the behavior of their classmates (Garcia-Rosales et al., 2021). We were also able to model genetic liability and maternal stress as moderators of maternal inflammation and infection. Finally, the large longitudinal population-based cohort used in the current study provides broadly generalizable results and ample statistical power to detect effect sizes of relevance for public health.

## 6. Conclusion

Maternal chronic inflammation, as measured by multiple cytokines as well as CRP, was not found to be a risk factor for child ADHD symptoms. Our findings contradict previous suggestions that prenatal inflammatory biomarkers such as CRP might be large, potentially causal precursors of ADHD. In contrast, the current study did find an association between infection during pregnancy and ADHD symptoms in children across childhood. The messaging of how impactful maternal infections are should be proportional to the small effect sizes observed, meaning most infections are unlikely to coincide with observable ADHD symptoms.

## Supplementary Material

Supplementary material

## Figures and Tables

**Table 1:** Characteristics of the analysis sample.

	Overall N = 6,555^1^	No ADHD symptomatology^1^ N = 5,834^2^	ADHD symptomatology N = 721^1^
**Child sex**			
Male	3,303 (50 %)	2,822 (48 %)	481 (67 %)
Female	3,252 (50 %)	3,012 (52 %)	240 (33 %)
**Mother’s age, years**	30.2 (5.0)	30.3 (4.9)	29.6 (5.6)
**Mother’s national origin**			
Dutch	3,444 (53 %)	3,056 (52 %)	388 (54 %)
Other Western	523 (8.0 %)	479 (8.2 %)	44 (6.1 %)
Other	2,588 (39 %)	2,299 (39 %)	289 (40 %)
**Mother’s educational level**			
Primary	573 (9.3 %)	498 (9.1 %)	75 (11 %)
Secondary	2,735 (45 %)	2,373 (44 %)	362 (52 %)
Higher	2,831 (46 %)	2,577 (47 %)	254 (37 %)
**Household income**			
<1,5996€	834 (17 %)	697 (16 %)	137 (23 %)
1,600 – 4,000€	2,405 (48 %)	2,097 (48 %)	308 (53 %)
>4,400€	1,714 (35 %)	1,572 (36 %)	142 (24 %)
**Mother living with/married to partner**	3,024 (49 %)	2,750 (50 %)	274 (40 %)
**Pre-pregnancy BMI**	23.6 (4.2)	23.6 (4.2)	23.9 (4.6)
**Parity**	0.6 (0.8)	0.6 (0.8)	0.5 (0.8)
**Prenatal stress score**	0.4 (0.4)	0.4 (0.4)	0.6 (0.4)
**Mother smoked during pregnancy**	1,014 (17 %)	859 (17 %)	155 (24 %)
**Maternal history of mental illness**	754 (14 %)	642 (14 %)	112 (20 %)

1 Below cut-off for ADHD symptomatology

2 Mean (SD); n (%)

3 Primary refers to education completed up to primary school; secondary up to lower and intermediate vocational training and higher up to higher vocational education and university.

## Data Availability

The data that has been used is confidential.

## Appendix A. Supplementary data

Supplementary data to this article can be found online at https://doi.org/10.1016/j.bbi.2025.106134.
