# Supplementary material for: Maternal infection – but not inflammation – is associated with attention-deficit/hyperactivity disorder symptoms during childhood: a population-based cohort study
Source: Brain Behav Immun. Author manuscript; Available in PMC 2026 Mar 15. (PMC12989210; doi:10.1016/j.bbi.2025.106134)
Supplement: Supplementary material [file NIHMS2153183-supplement-Supplementary_material.docx]

Maternal infection – but not inflammation – is associated with attention-deficit/hyperactivity disorder symptoms during childhood: a population-based cohort study

**Supplementary materials**

**Supplementary Figure 1.** Directed acyclic graph of confounders, mediators and covariates for the association between inflammation, infection and ADHD symptoms.

**
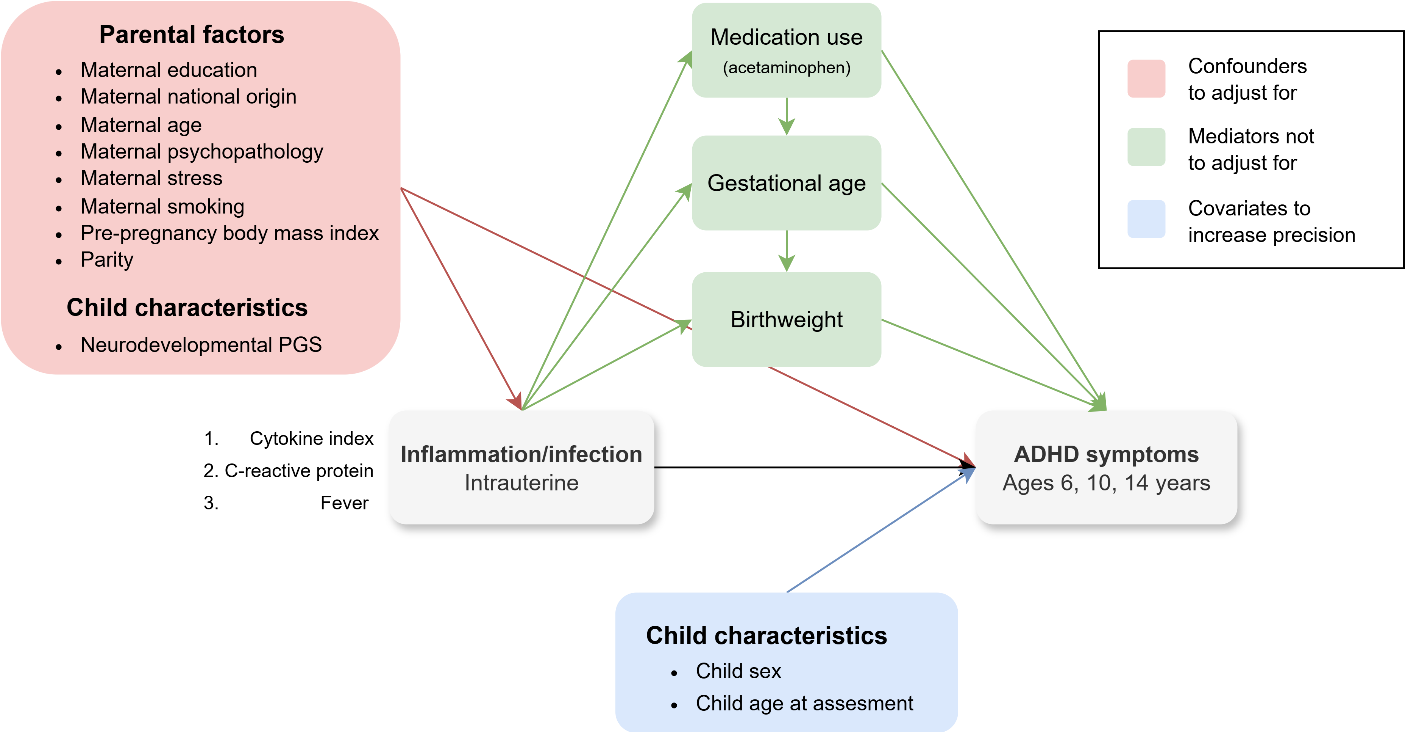
**

Note: no mediation analysis was carried out, mediators are depicted to inform adjustment strategy

| **Supplementary Table 1.** Comparison of participants in the final analysis sample to those not participating due to missing data on ADHD symptoms (N = 2,072) or inflammation markers (N = 1,207). | | | | |
| --- | --- | --- | --- | --- |
|  | **Included in analysis**  N = 6,555*^1^* | **Non in analysis sample**  N = 3,279*^1^* | **Group difference effect size**^2^ | **p-value** |
| **Child sex** |  |  | 0.96 | 0.412 |
| Male | 3,303 (50%) | 1,602 (51%) |  |  |
| Female | 3,252 (50%) | 1,520 (49%) |  |  |
| **Mother's age, years** | 30.2 (5.0) | 29.2 (6.0) | 0.19 | <0.001 |
| **Mother's national origin** |  |  |  |  |
| Dutch | 3,444 (53%) | 1,129 (34%) | 0.47 | <0.001 |
| Other Western | 523 (8%) | 247 (8%) | 0.94 | 0.498 |
| Other | 2,588 (39%) | 1,903 (58%) | 2.12 | <0.001 |
| **Mother's education** |  |  |  |  |
| Primary | 573 (9%) | 368 (15%) | 1.73 | <0.001 |
| Secondary | 2,735 (45%) | 1,219 (50%) | 1.25 | <0.001 |
| Higher | 2,831 (46%) | 845 (35%) | 0.62 | <0.001 |
| **Pre-pregnancy BMI** | 23.6 (4.2) | 23.8 (5) | -0.06 | 0.841 |
| **Parity** | 0.6 (0.8) | 0.8 (1.0) | -0.17 | <0.001 |
| **Prenatal stress score** | 0.4 (0.4) | 0.6 (0.4) | -0.41 | <0.001 |
| **Smoking during pregnancy** |  |  |  |  |
| Never smoked during pregnancy | 4,310 (74%) | 1,771 (73%) | 0.96 | 0.422 |
| Smoked until pregnancy was known | 525 (9%) | 182 (7%) | 0.82 | 0.027 |
| Continued smoking in pregnancy | 1,014 (17%) | 479 (20%) | 1.17 | 0.011 |
| **Maternal history of mental illness** |  |  | 1.77 | <0.001 |
| None | 4,557 (86%) | 1,038 (77%) |  |  |
| Pre-existing condition | 754 (14%) | 304 (23%) |  |  |
| **IL1β**^2^ | 1.4 (0.5) | 1.4 (0.5) | 0.04 | 0.010 |
| **IL6**^2^ | 0.6 (1.0) | 0.6 (1.0) | 0.02 | 0.394 |
| **IL17A**^2^ | 3.2 (0.6) | 3.2 (0.6) | 0.07 | 0.015 |
| **IFNγ**^2^ | 2.7 (0.6) | 2.6 (0.6) | 0.08 | 0.025 |
| **IL23**^2^ | 7.1 (0.7) | 7.0 (0.7) | 0.07 | 0.001 |
| **Cytokine composite index** | 0.0 (1.8) | -0.1 (1.9) | 0.07 | 0.003 |
| **CRP**^2^ | 1.4 (0.8) | 1.5 (0.9) | -0.10 | <0.001 |
| **Fever count** | 0.2 (0.6) | 0.2 (0.6) | -0.01 | 0.557 |
| **Infection score** | 3.1 (2.3) | 3.1 (2.5) | -0.01 | 0.485 |
| *^1^* n (%); Mean (SD)  *^2^*  Presented on log scale | | | | |
| ^3^ Cohen’s d; Odds ratio (ref=Included) | | | | |

**
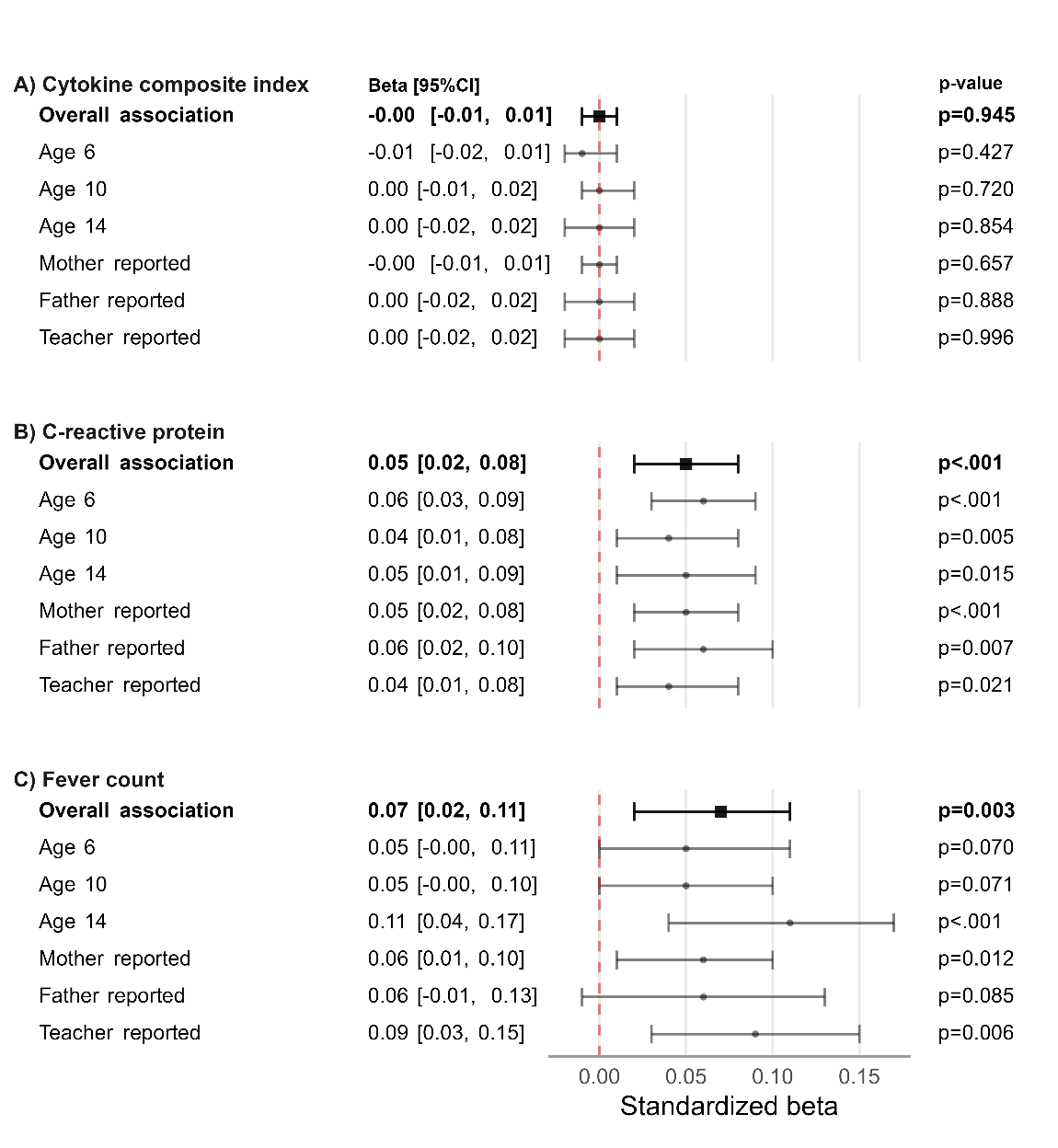
Supplementary Figure 2.** Unadjusted overall, age-specific, and informant-specific standardized associations between maternal inflammation/infection and ADHD symptoms. Overall associations represent weighted averages marginalized over all time and informant assessments.

| **Supplementary Table 2.** Overall associations from the sensitivity analyses where the main outcome ADHD symptoms as measured via the CBCL was replaced with a secondary outcome, the ADHD index measured via the Conners’ Parent Rating Scale–Revised (CPRS-R). | | |
| --- | --- | --- |
|  | **Overall association [95%CI]*^1^*** | **p-value** |
| **Cytokine composite index** | 0.00 [-0.01, 0.02] | 0.574 |
| **C-reactive protein** | 0.01 [-0.03, 0.04] | 0.679 |
| **Fever count** | 0.04 [-0.02, 0.11] | 0.204 |
| *^1^* Adjusted for maternal age, maternal education, maternal national origin, maternal psychopathology, maternal smoking during pregnancy, maternal body mass index before pregnancy, parity, child age and child sex | | |

| **Supplementary Table 3.** Overall odds ratios from sensitivity analyses where the main ADHD symptoms outcome was replaced with dichotomized cut-offs for clinical ADHD symptoms. Cut-offs were placed above the top 5% of ADHD symptoms for each measurement. | | |
| --- | --- | --- |
|  | **Overall odds ratio [95%CI]*^1^*** | **p-value** |
| **Cytokine composite index** | 1.02 [0.91, 1.15] | 0.697 |
| **C-reactive protein** | 0.99 [0.76, 1.30] | 0.943 |
| **Fever count** | 1.15 [0.79, 1.65] | 0.467 |
| *^1^* Adjusted for maternal age, maternal education, maternal national origin, maternal psychopathology, maternal smoking during pregnancy, maternal body mass index before pregnancy, parity, child age and child sex | | |


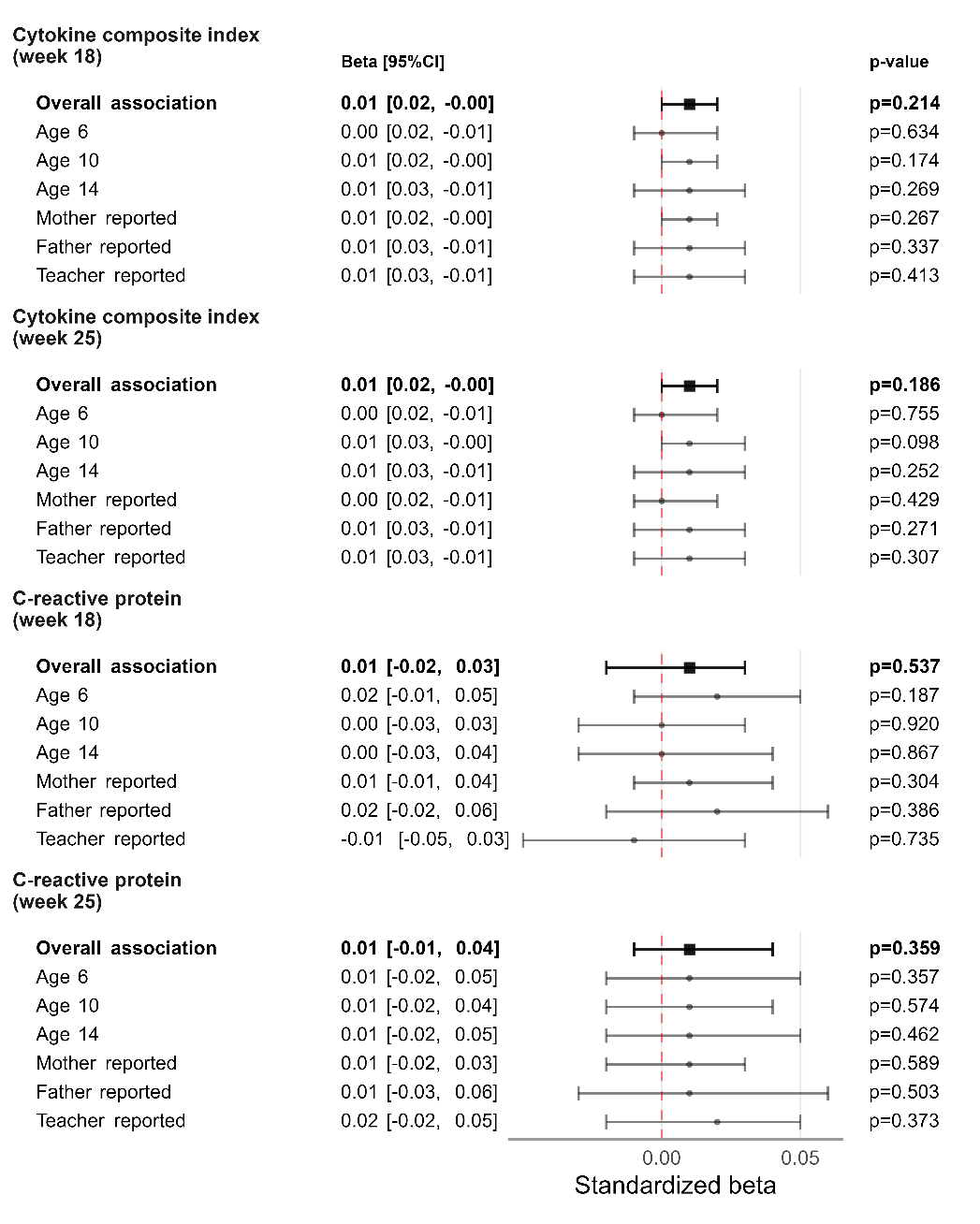
**Supplementary Figure 3.** Overall, age-specific and informant-specific standardized associations between maternal inflammation measured at average week 18, average week 25 and attention-deficit/hyperactivity problems.

Note: All estimates adjusted for maternal age, maternal education, maternal national origin, maternal psychopathology, maternal smoking during pregnancy, maternal body mass index before pregnancy, parity, child age and child sex

| **Supplementary Table 4.** Overall associations between trimester-specific exposure to fever and ADHD symptoms, summarized across all timepoints. Associations presented as standardized betas. | | |
| --- | --- | --- |
|  | **Overall association [95%CI]*^1^*** | **p-value** |
| **Fever in first trimester** | 0.06 [-0.01, 0.14] | p=0.080 |
| **Fever in second trimester** | 0.07 [-0.02, 0.17] | p=0.124 |
| **Fever in third trimester** | 0.09 [-0.003, 0.18] | p=0.058 |
| *^1^* Adjusted for maternal age, maternal education, maternal national origin, maternal psychopathology, maternal stressful events during pregnancy, maternal smoking during pregnancy, maternal body mass index before pregnancy, parity, child polygenic risk score for neurodevelopmental disorders, child age and child sex | | |

| **Supplementary Table 5.** Overall association between individual cytokine levels and ADHD symptoms, summarized across all time points. Associations presented as standardized betas. | | | | |
| --- | --- | --- | --- | --- |
|  | CBCL | | CPRS-R | |
|  | **Overall association [95%CI]*^2^*** | **p-value** | **Overall association [95%CI]*^2^*** | **p-value** |
| **IL-1β** | 0.01 [-0.03, 0.05] | 0.578 | 0.00 [-0.06, 0.07] | 0.924 |
| **IL-6** | 0.01 [-0.01, 0.03] | 0.182 | 0.01 [-0.02, 0.04] | 0.405 |
| **IL-17** | 0.01 [-0.02, 0.05] | 0.415 | 0.00 [-0.05, 0.06] | 0.875 |
| **IL-23** | 0.01 [-0.01, 0.04] | 0.346 | 0.02 [-0.02, 0.06] | 0.351 |
| **IFN-γ** | 0.01 [-0.02, 0.05] | 0.396 | 0.01 [-0.04, 0.07] | 0.582 |
| *^1^* Adjusted for maternal age, maternal education, maternal national origin, maternal psychopathology, maternal stressful events during pregnancy, maternal smoking during pregnancy, maternal body mass index before pregnancy, parity, child polygenic risk score, child age and child sex  ADHD = Attention-deficit/hyperactivity disorder; CBCL = Child Behavior Checklist; CPRS-R = Conners’ Parental Rating Scale – Revised | | | | |

**
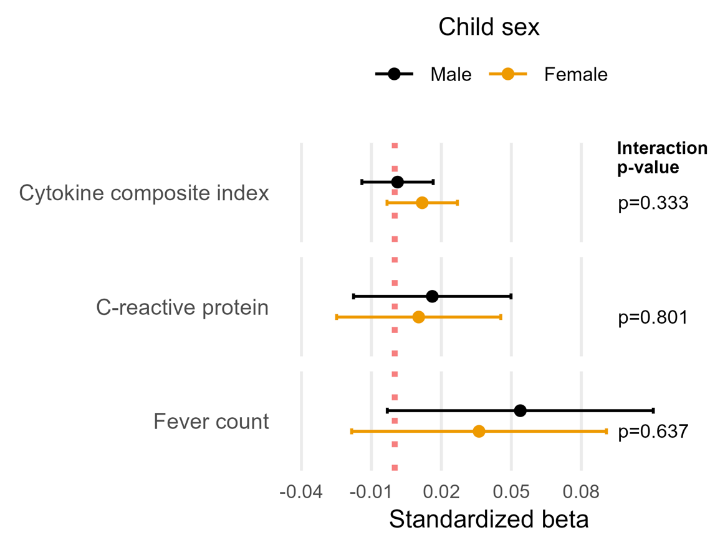
Supplementary Figure 4.** Associations between maternal inflammation/infection and ADHD symptoms moderated by child sex. P-values obtained from interaction term between each exposure and child sex.

Note: All estimates adjusted for maternal age, maternal education, maternal national origin, maternal psychopathology, maternal smoking during pregnancy, maternal body mass index before pregnancy, parity, child age and child sex

**Supplementary Figure 5.** Results from sensitivity analysis in which the fever exposure has been replaced with a broader self-report infection score.

**
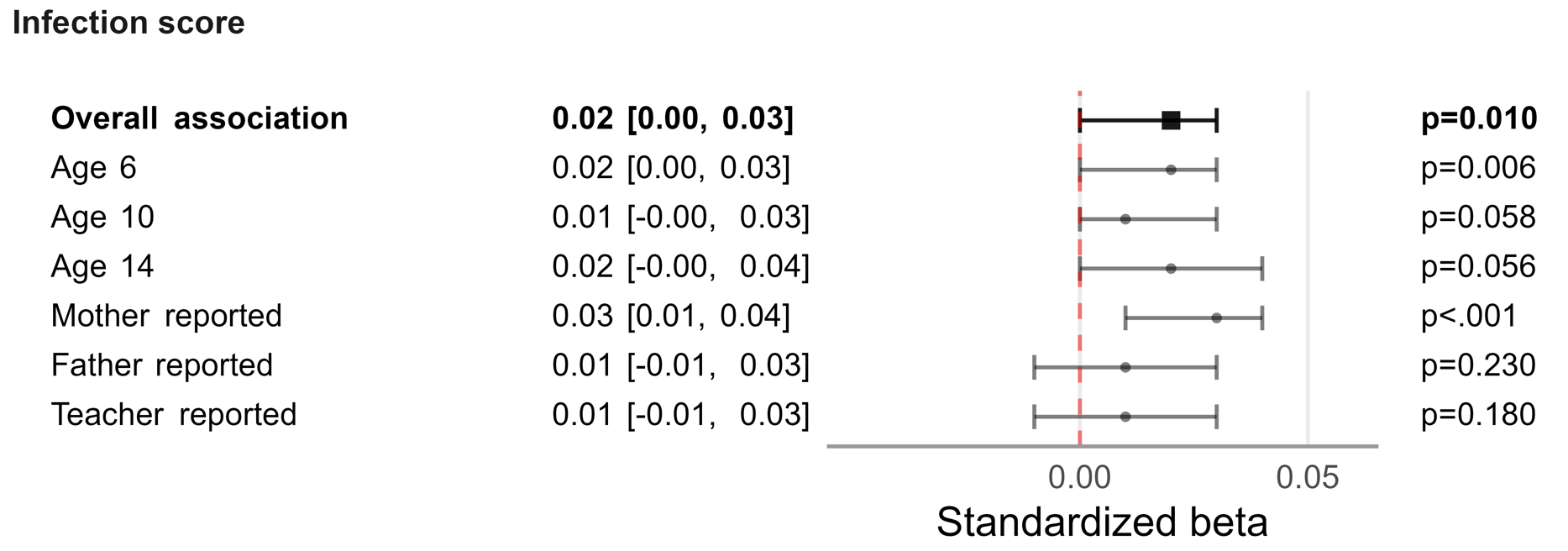
**

Note: Infection score included 1) any upper respiratory infections; 2) lower respiratory infections; 3) gastrointestinal infections; 4) cystitis/pyelitis; 5) dermatitis; 6) eye infections; 7) herpes zoster; 8) sexually transmitted diseases, and (9) flu. All estimates adjusted for maternal age, maternal education, maternal national origin, maternal psychopathology, maternal smoking during pregnancy, maternal body mass index before pregnancy, parity, child age and child sex.

**Supplementary Figure 6.** Results from sensitivity analysis in which the neurodevelopmental PGS moderation analysis is conducted only within children from European background.

**
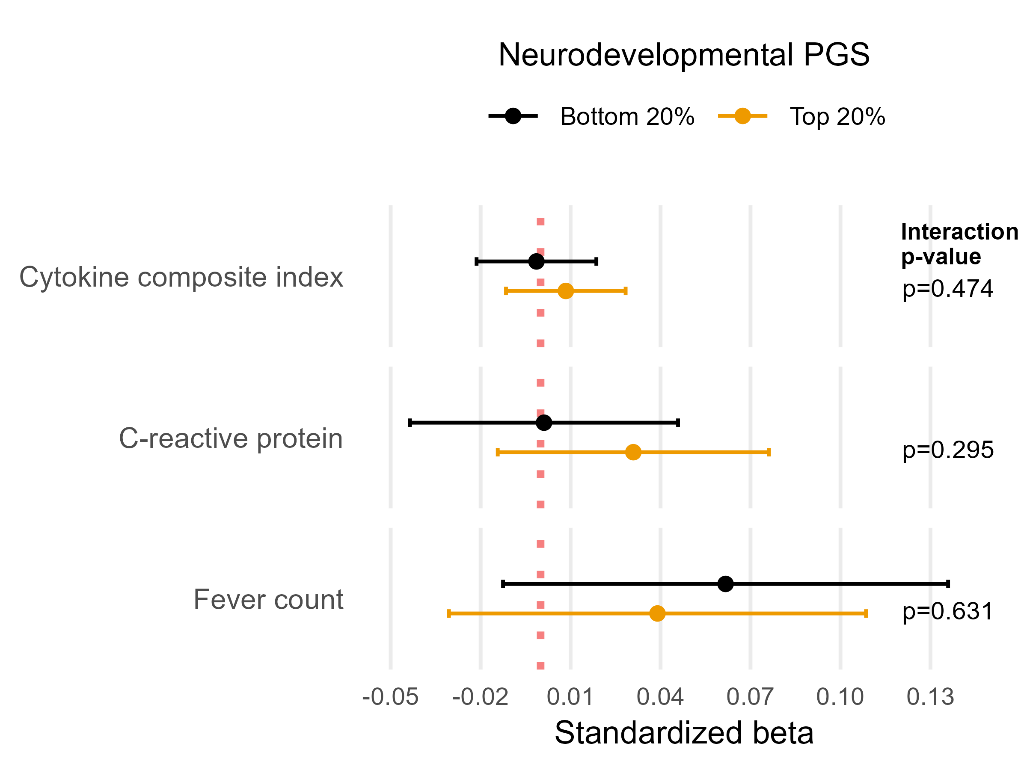
**

All estimates adjusted for maternal age, maternal education, maternal psychopathology, maternal smoking during pregnancy, maternal body mass index before pregnancy, parity, child age and child sex.

**Supplementary Figure 7.** Associations between maternal inflammation/infection and ADHD symptoms moderated by maternal acetaminophen use during pregnancy. P-values obtained from interaction term between each exposure and acetaminophen use during pregnancy.

**
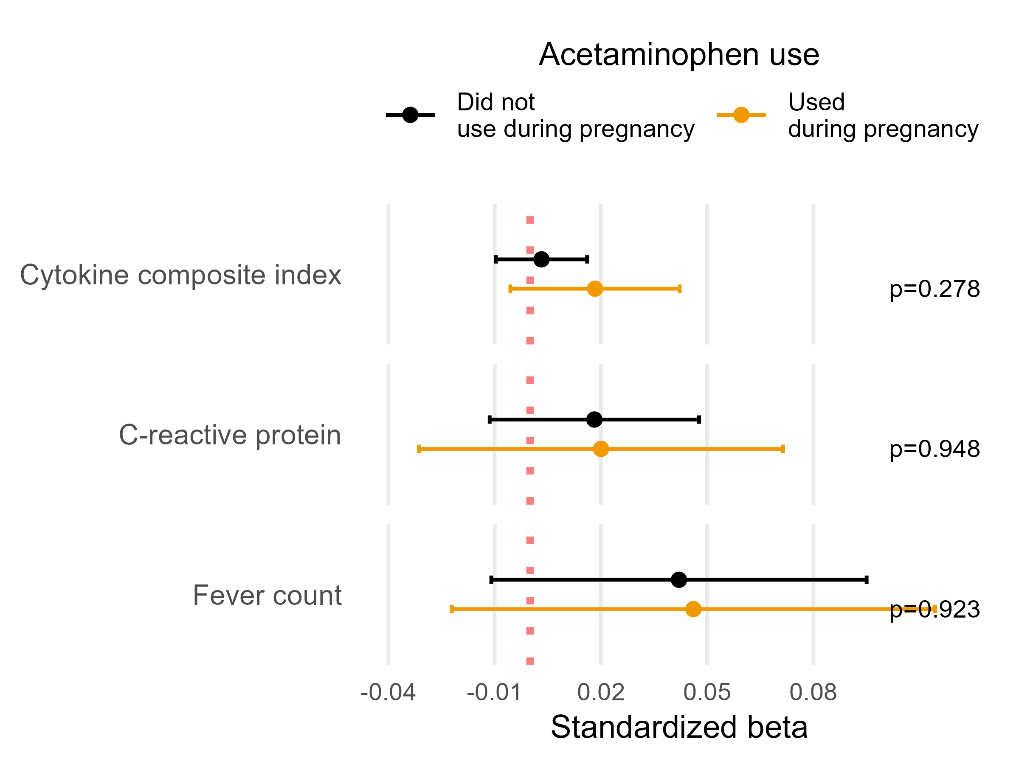
**

All estimates adjusted for maternal age, maternal education, maternal psychopathology, maternal smoking during pregnancy, maternal body mass index before pregnancy, parity, child age and child sex.
